# Fulvestrant and everolimus efficacy after CDK4/6 inhibitor: a prospective study with circulating tumor DNA analysis

**DOI:** 10.1038/s41388-024-02986-6

**Published:** 2024-02-27

**Authors:** Antoine Vasseur, Luc Cabel, Caroline Hego, Wissam Takka, Olfa Trabelsi Grati, Benjamin Renouf, Florence Lerebours, Delphine Loirat, Etienne Brain, Paul Cottu, Marie-Paule Sablin, Jean-Yves Pierga, Céline Callens, Shufang Renault, François-Clément Bidard

**Affiliations:** 1 Department of Medical Oncology, Institut Curie, Paris & Saint-Cloud, France; 2 Circulating Tumor Biomarkers Laboratory, INSERM CIC BT-1428, Institut Curie, Paris, France; 3 Department of Genetics, Institut Curie, Paris Sciences & Lettres University, Paris, France; 4 Clinical Research Unit, Institut Curie, Paris, France; 5 Université Paris Cité, Paris, France; 6 UVSQ, Paris-Saclay University, Saint Cloud, France

**Keywords:** Breast cancer, Prognostic markers

## Abstract

In a prospective study (NCT02866149), we assessed the efficacy of fulvestrant and everolimus in CDK4/6i pre-treated mBC patients and circulating tumor DNA (ctDNA) changes throughout therapy. Patients treated with fulvestrant and everolimus had their ctDNA assessed at baseline, after 3–5 weeks and at disease progression. Somatic mutations were identified in archived tumor tissues by targeted NGS and tracked in cell-free DNA by droplet digital PCR. ctDNA detection was then associated with clinicopathological characteristics and patients’ progression-free survival (PFS), overall survival (OS) and best overall response (BOR). In the 57 included patients, median PFS and OS were 6.8 (95%CI [5.03–11.5]) and 38.2 (95%CI [30.0-not reached]) months, respectively. In 47 response-evaluable patients, BOR was a partial response or stable disease in 15 (31.9%) and 11 (23.4%) patients, respectively. Among patients with trackable somatic mutation and available plasma sample, N = 33/47 (70.2%) and N = 19/36 (52.8%) had ctDNA detected at baseline and at 3 weeks, respectively. ctDNA detection at baseline and *PIK3CA* mutation had an adverse prognostic impact on PFS and OS in multivariate analysis. This prospective cohort study documents the efficacy of fulvestrant and everolimus in CDK4/6i-pretreated ER + /HER2- mBC and highlights the clinical validity of early ctDNA changes as pharmacodynamic biomarker.

## Introduction

Breast cancer is the most common cancer among women globally, with an estimated 2.3 million new cases and 685 000 deaths in 2020 in the world [1]. Recently, the prognosis of estrogen receptor-positive/HER2-negative (ER + /HER2-) metastatic breast cancer (mBC) has been improved following the development of cyclin-dependent kinase 4/6 inhibitors (CDK 4/6i). Three CDK4/6i (palbociclib, ribociclib and abemaciclib) [2–4] in combination with aromatase inhibitors [5] or fulvestrant (a selective estrogen receptor degrader) [6] have extended progression-free survival (PFS) and overall survival (OS) [7]. These drugs became standard of care as first line treatment for ER + /HER2- mBC patients, in the absence of visceral crisis.

Upon progression on first line CDK4/6i and endocrine therapy (ET), the combination of fulvestrant with everolimus (targeting the PI3K/AKT/mTOR pathway) is recognized as a valid second line treatment option by National Comprehensive Cancer Network (NCCN) and European Society for Medical Oncology (ESMO) [8] guidelines for ER + /HER2- mBC. The underlying evidence mostly consists in two randomized phase 2 trials, PrE0102 [9] and MANTA [10], both conducted in ER + /HER2- mBC patients whose mBC has become resistant to aromatase inhibitors. In these studies, patients treated with fulvestrant and everolimus showed a median PFS of 10.3 and 12.3 months, compared to 5.1 and 5.4 months with fulvestrant alone, respectively. However, these efficacy data were obtained without prior CDK4/6i therapy and no biomarker associated with fulvestrant and everolimus efficacy has been validated so far.

Circulating tumor DNA (ctDNA), a fraction of total cell-free circulating DNA (cfcDNA), has emerged in the last decade as a promising biomarker for diagnosis, prognosis, and monitoring treatment efficacy in different cancer types [11–13]. In ER + /HER2- mBC treated by ET and CDK4/6i, our group and others have previously shown that early ctDNA decrease was a prognostic factor for PFS [14–16] and the efficiency of palbociclib and fulvestrant can be monitored by serial analyses of ctDNA [16].

This study was set up to prospectively document the efficacy of the everolimus plus fulvestrant combination after progression on CDK4/6i and, with the use of personalized droplet digital PCR (ddPCR) assays, to investigate the clinical validity of ctDNA early changes as pharmacodynamic marker in ER + /HER2- mBC patients.

## Results

### Patient characteristics

From February 2018 to October 2021, 57 patients with ER + /HER2- mBC after progression on a CDK4/6i were enrolled in this study. The main characteristics of the 57 patients are as follows: median age was 57 years (range [36–79]), 30 (52.6%) patients had ≥3 metastatic sites and 33 (57.9%) had visceral metastases. Most patients (N = 48, 84.2%) had received only one prior line of systemic therapy, ET (aromatase inhibitor) and CDK4/6i. Only one patient has already received fulvestrant in the previous lines of metastatic disease. Patient characteristics are shown in Table 1.

**Table 1:** Patients’ characteristics.

Characteristics	Number of patients N = 57	%	ctDNA at baseline N = 47			ctDNA at 3–5 weeks N = 36		
			Positive N = 33	Negative N = 14	P value	Positive N = 19	Negative N = 17	P value
Age								
<60	35	61.4	20	8	0.80	9	12	0.20
≥60	22	38.6	13	6		10	5	
Performance status								
0	42	73.7	21	12	0.20	12	13	0.40
1	15	26.3	12	2		7	4	
Primary tumor grade								
1–2	48	84.2	29	12	>0.90	17	14	>0.90
3	8	14.0	4	1		2	2	
Missing	1	1.8	0	1		0	1	
Primary Ki67								
≤20%	20	35.1	11	4	>0.90	8	3	0.06
>20%	28	49.1	18	7		8	13	
Missing	9	15.8	4	3		3	1	
Neoadjuvant chemotherapy								
Yes	24	42.1	15	5	0.50	8	8	0.70
No	33	57.9	18	9		11	9	
De novo stage IV								
Yes	23	40.4	12	7	0.40	8	7	0.80
No	34	59.6	21	7		11	9	
Number of metastatic sites								
<3	27	47.4	13	9	0.12	7	12	**0.04**
≥3	30	52.6	20	5		12	5	
Bone only metastases								
Yes	15	26.3	8	4	0.70	4	7	0.20
No	42	73.7	25	10		15	10	
Visceral metastases								
Yes	33	57.9	19	8	>0.90	11	8	0.50
No	24	42.1	14	6		8	9	
Prior number of lines of therapy*								
=1	48	84.2	28	13	0.70	16	16	0.60
>1	9	15.8	5	1		3	1	
*PIK3CA* mutation								
Yes	25	43.9	16	9	0.30	12	8	0.30
No	22	38.6	17	5		7	9	
Missing	10	17.5	0	0		0	0	
Length of prior CDK4/6i (months)								
Median (range)	13.6	(12.7-16.6)						

^*^For metastatic disease.

**Fig. 1:**
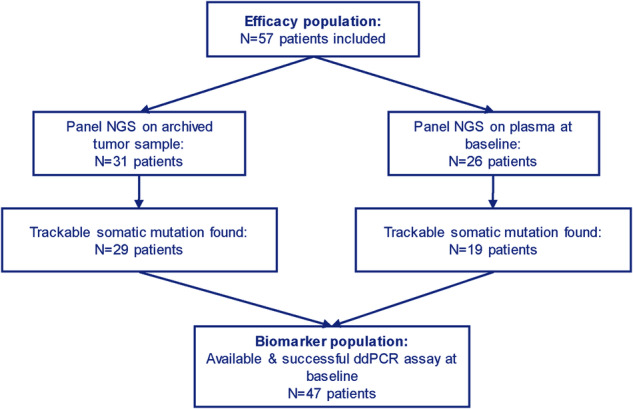
Workflow of the study. ER + : estrogen receptor positive; mBC: metastatic breast cancer; NGS: next-sequencing generation; ddPCR: droplet digital PCR.

Of these 57 patients, 31 patients had archived tumor tissue available; 29 of them displayed at least one somatic mutation in tumor DNA by targeted NGS. Further targeted NGS, performed on baseline cfcDNA in those without available tissue DNA, identified another 19 patients with detectable mutations (Fig. 1). Finally, 47 patients with available ddPCR assays were tested for ctDNA levels before and after treatment targeting the identified mutation (*PIK3CA*_mut_: N = 25; *ESR1*_mut_: N = 7; *TP53*_mut_: N = 4; *AKT*_mut_: N = 2; *GATA3*_mut_: N = 2; *CUX1*_mut_: N = 1; *PTEN*_mut_: N = 1; *APC*_mut_: N = 1; *ATM*_mut_: N = 1; *RET*_mut_: N = 1; *TRAPP*_mut_: N = 1; *KRAS*_mut_: N = 1; Supplementary Table 1). Of the 25 patients identified with a *PIK3CA* mutation (including the one with a *PIK3CA* variant of unknown significance), 20 patients had tumor tissue DNA available, and 5 patients were tested with baseline cfcDNA.

### Patients’ outcome

**Fig. 2:**
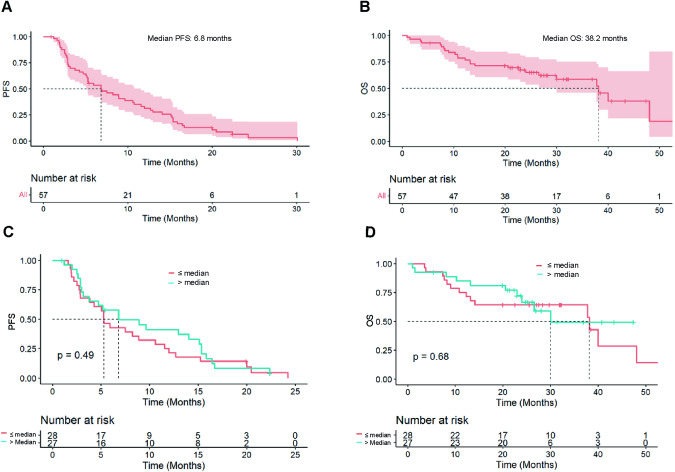
Progression-free survival and overall survival in patients treated with everolimus and fulvestrant. In the whole population (**A**, **B**) and according to previous palbociclib treatment duration (above or under/equal to median PFS (**C**, **D**)). PFS progression-free survival, OS overall survival.

With a median follow-up of 29.7 months, median PFS and OS with everolimus plus fulvestrant post CDK4/6i (palbociclib) were 6.8 (95%CI [5.03–11.5]) months and 38.2 (95%CI [30.0-not reached (NR)]) months, respectively (Fig. 2A, B). Among the 47 patients with measurable disease at baseline, 15 (31.9%) experienced a partial response (PR). Eleven (23.4%) patients had SD and 21 (44.7%) had PD as BOR (Supplementary Table 2).

We assessed whether the duration of treatment with palbociclib (using median PFS as threshold, 13.6 months) affected survival in patients treated with everolimus and fulvestrant. Interestingly, there was no effect on survival with everolimus plus fulvestrant (for PFS: median PFS of 6.8 (95%CI [3.75–15.3]) months for patients with longer exposure versus 5.3 (95%CI [3.82–11.5]) months, p = 0.49 and for OS: median OS of 30.0 (95%CI [24.0-NR]) months for patients with longer exposure versus 38.2 (95%CI [14.1-NR]) months, p = 0.68 (Fig. 2C, D)). Patients with a long duration of response to ET + palbociclib treatment in the previous line (PFS > 24 months) had a numerically longer PFS than those with a PFS ≤ 24 months (median PFS: 13.0 months versus 5.3 months, p = 0.16). Patients with a rapid progression to ET + palbociclib treatment (PFS < 6 months) had a shorter PFS, but it was not significantly associated (4.5 months versus 6.8 months for patients with a PFS ≥ 6 months, p = 0.41) (Supplementary Figure 1). We also evaluated survival in the 25 patients with *PIK3CA* mutation. A shorter median PFS and OS was observed (for PFS: 3.4 (95%CI [2.83-8.91]) months versus 9.9 (95%CI [5.30-15.33]) months, p = 0.008 and for OS 22.9 (95%CI [10.8-NR]) months versus 40.0 (95%CI [30.0-NR]) months, p = 0.06, respectively) (Supplementary Figure 2A, B). The prognostic impact of other (i.e. non-*PIK3CA*) mutations was also investigated. No significant difference was observed on PFS with *ESR1* mutation (N = 7/47, p = 0.15) (Supplementary Fig. 3). For *TP53* (N = 4) or *AKT1* (N = 2) mutation, due to the limited number of patients included, the analysis was lack of statistical power and data was not shown.

### Safety

The most common adverse events of any grade occurring in at least 25% of patients were mucositis (N = 39/57, 68.4%), asthenia (N = 27/57, 47.4%), decreased weight (N = 18/57, 31.6%), rash (N = 18/57, 31.6%), hypercholesterolemia (N = 16/57, 28.1%), hypertriglyceridemia (N = 15/57, 26.3%), alanine aminotransferase increased (N = 15/57, 26.3%) and dyspnea (N = 15/57, 26.3%). Grade 3 or 4 adverse events were mucositis (N = 6/57, 10.5%), hypertriglyceridemia (N = 2/57, 3.5%), neutropenia (N = 1/57, 1.8%), decreased body weight (N = 1/57, 1.8%) and pneumonitis (N = 1/57, 1.8%) (Table 2). Ten patients (17.5%) discontinued everolimus due to toxicity and 17 (29.8%) patients required a dose reduction.

**Table 2:** Adverse events with everolimus plus fulvestrant.

Adverse events	All grades		Grade 3 and 4	
	Number of patients N = 57	%	Number of patients N = 57	%
Hyperglycemia	13	22.8	0	0
Mucositis	39	68.4	6	10.5
Pneumonitis	9	15.8	1	1.8
Decreased weight	18	31.6	1	1.8
Nausea	8	14.0	0	0
Rash	18	31.6	0	0
Diarrhea	13	22.8	0	0
Constipation	6	10.5	0	0
hypercholesterolemia	16	28.1	0	0
Neutropenia	4	7.0	1	1.8
Hypertriglyceridemia	15	26.3	2	3.5
Alanine aminotransferase increased	15	26.3	0	0
Asthenia	27	47.4	0	0
Dyspnea	15	26.3	0	0

### ctDNA detection and prognostic value

**Fig. 3:**
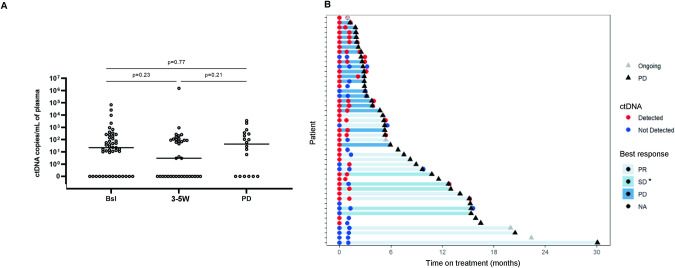
ctDNA detection at different time points. **A** ctDNA levels at different time points quantified by ddPCR. Each dot represents the level of ctDNA of a patient measured. Mann–Whitney test was used to compare ctDNA levels at different time points. **B** Swimmer plot showing the dynamic changes of ctDNA levels, best overall response and timing of clinical relapse. Each bar represents one patient. Red circles indicate ctDNA detection and blue circles indicate no ctDNA detection. The black triangle indicates disease progression (PD). *SD ≥ 6 months. Bsl: baseline; 3–5 W: 3–5 weeks, PD progressive disease, PR partial response, SD stable disease, NA not available.

Among the 47 patients with a trackable mutation, N = 33/47 patients (70.2%) showed detectable ctDNA at baseline with a median allelic frequency of 1.62% (range (0.0-52.6)) (Fig. 3A and Supplementary Table 3). Three weeks after treatment, matched plasma samples were available for 36 patients, of which N = 19/36 patients (52.8%) were ctDNA-positive with a median allelic frequency of 0.2% (range [0.0-55.1]). Nine patients had no ctDNA detected at both baseline and 3–5 W. Seventeen patients (47.2%) showed a ctDNA decrease at 3–5 W, including 8 patients with ctDNA clearance (22.2%), while 10 patients showed an increase of ctDNA (50.0%). At the time of tumor progression, N = 12/18 (66.7%) patients had ctDNA detected with a median allelic frequency of 1.9% (range [0.0-42.5]) (Fig. 3A, B and Supplementary Table 3). No significant association was found for ctDNA levels between the different time points (Fig. 3A). The timing of clinical relapse of the different patients is shown in Fig. 3B.

**Fig. 4:**
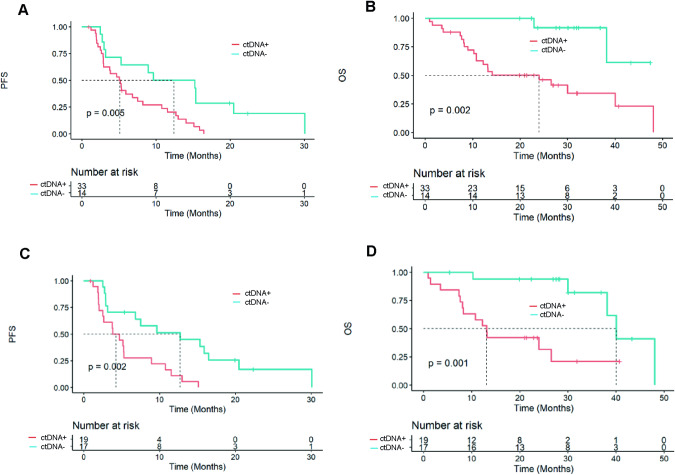
Prognostic value of ctDNA detection at different time points. Progression-free survival and overall survival according to ctDNA detection at baseline (**A**, **B**) and 3 weeks (**C**, **D**). PFS progression-free survival, OS overall survival.

The prognostic value of ctDNA detection at baseline and 3–5 W was then investigated. Both ctDNA detection at baseline and 3–5 W were associated with shorter median PFS (5.1 months versus 12.4 months, HR = 2.04, 95%CI [1.20–3.45], p = 0.005 and 4.3 months versus 12.7 months, HR = 2.56, 95%CI [1.41–4.76], p = 0.002, respectively). OS was also shorter in patients with both ctDNA detection at baseline and 3–5 W (24 months versus NR months, HR = 3.85, 95%CI [1.39–10.99], p = 0.002 and 13.2 months versus 40.0 months, HR = 3.85, 95%CI [1.56–10.00], p = 0.001, respectively) (Fig. 4A–D). Among patients with ctDNA detected at baseline, ctDNA levels was not significantly associated with PFS (ctDNA > vs < median copy number, HR = 1.40, 95%CI [0.69-3.00], p = 0.33) (Supplementary Table 4). Among the patients with *PIK3CA* mutation, ctDNA detection at baseline showed no prognostic value (Supplementary Figure 4).

### Early ctDNA dynamics as pharmacodynamic marker

**Fig. 5:**
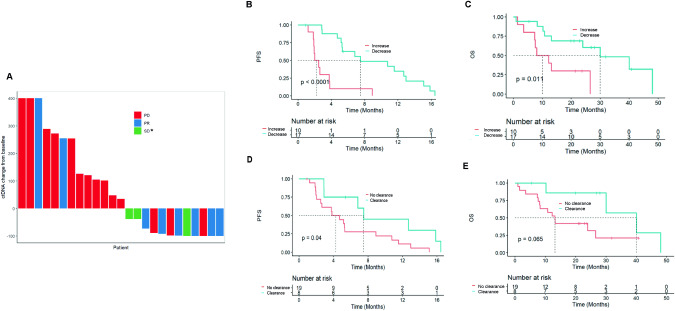
Tumor response and prognostic value of early ctDNA variations. Tumor response according to ctDNA change from baseline (**A**). *SD ≥ 6 months. Progression-free survival and overall survival according to early ctDNA variation (**B**, **C**) and ctDNA clearance (**D**, **E**). PD progressive disease, PR partial response, SD stable disease, PFS progression-free survival; OS: overall survival.

Among 27 patients with positive ctDNA detection at baseline and/or 3–5 W, 25 patients were available for analysis of correlation with the first tumor assessment (one patient was not followed up and the other patient had no measurable disease according to RECIST criteria). The majority of patients with an increase in ctDNA between baseline and 3–5 W had disease progression (N = 11/13, 84.6%) at the first tumor assessment (2 patients had a PR). Among patients with a decrease in ctDNA, 3 patients (N = 3/12, 25%) had PD at the first tumor assessment (6 patients had a PR and 3 patients had SD) (Fig. 5A). Overall, using a ‘ctDNA decreased at 3–5 W’ vs ‘ctDNA increased at 3–5 W’ threshold, ctDNA changes accuracy to predict disease progression at first tumor assessment was 80.0%, with N = 20 correct predictions out of 25 assessable patients.

In terms of survival, patients with a ctDNA decrease at 3–5 W (regardless of threshold) had a significantly longer median PFS (7.5 months vs 2.2 months; HR = 0.15, 95%CI [0.06-0.41], p < 0.0001) and median OS (40.0 months vs 12.2 months; HR = 0.27, 95%CI [0.09-0.79], p = 0.011) (Fig. 5B, C). The same trend was observed for ctDNA clearance at 3–5 W: longer median PFS (7.5 months vs 4.2 months; HR = 0.36, 95%CI [0.136-0.99], p = 0.04) and median OS (40.0 months vs 13.2 months; HR = 0.31, 95%CI [0.09-1.10], p = 0.065) were found in patients with ctDNA clearance (Fig. 5D, E). We explored two other thresholds of ctDNA changes, -30% and -50% in copy number at 3–5 W: both were associated with PFS (HR = 0.15, 95%CI [0.06-0.41], p < 0.0001 and HR = 0.34, 95%CI [0.15-0.81], p = 0.011, respectively) and OS (HR = 0.27, 95%CI [0.09-0.79], p = 0.011 and HR = 0.27, 95%CI [0.07-0.84], p = 0.017, respectively) (data not shown).

### Univariate and multivariate analysis

Finally, univariate and multivariate proportional hazards models were performed to evaluate the prognostic value of prospectively collected clinicopathological factors combined with ctDNA detection at different time points. As shown in Supplementary Table 4, *PIK3CA* mutation detection (in a subgroup of N = 25 (43.8%) patients with *PIK3CA* mutations), ctDNA detection at baseline and 3–5 W, ctDNA decrease between baseline and 3–5 W, and ctDNA clearance at 3–5 W were significantly associated with PFS and OS in univariate analysis (except for the presence of *PIK3CA* mutation and ctDNA clearance at 3–5 W, where there was no significant difference in OS), while age and performance status were only associated with OS (HR = 1.00, 95%CI [1.00–1.10], p = 0.03 and HR = 2.90, 95%CI [1.20-6.90], p = 0.02).

In multivariate analysis, baseline ctDNA positivity and *PIK3CA* mutation were found as independent prognostic factors for PFS and OS (for PFS, HR = 2.36, 95%CI [1.38-4.04], p = 0.002; HR = 2.67, 95%CI [1.39-5.10], p = 0.003; for OS, HR = 6.33, 95%CI [2.07-19.36], p = 0.001; HR = 4.58, 95%CI [1.57-13.35], p = 0.005, respectively) (Table 3).

**Table 3:** Multivariate analysis for progression-free survival and overall survival.

Cox multivariate analysis		
Variable	HR (95%CI)	*p* value
Progression-free survival		
Bone only metastases	0.47 (0.21-1.04)	0.06
* PIK3CA* mutation	2.67 (1.39-5.10)	**0.003**
Baseline ctDNA detection	2.36 (1.38-4.04)	**0.002**
Overall survival		
Performance status	1.23 (0.40-3.82)	0.72
Age	1.02 (0.98-1.07)	0.39
* PIK3CA* mutation	4.58 (1.57-13.35)	**0.005**
Baseline ctDNA detection	6.33 (2.07-19.36)	**0.001**

*HR* hazard ratio, *CI* confidence interval.

## Discussion

To our knowledge, this is the first prospective study to evaluate the efficacy of everolimus in combination with fulvestrant after progression on CDK4/6i in ER + /HER2- mBC. We document a median PFS of 6.8 months with everolimus and fulvestrant. This median PFS is apparently shorter than that reported in the PrE0102 and MANTA trials, which included patients not pre-treated with CDK4/6i [9, 10]. A similar shortening of second line PFS in CDK4/6i pretreated patients was previously observed with the fulvestrant-alpelisib combination, with median PFS of 11.0 months when given to CDK4/6i-naïve (SOLAR-1 trial [17]) versus 7.3 (BYLieve trial [18]) and 5.3 (French early access program [19]) months in CDK4/6i-pretreated mBC patients. In the post CDK4/6i setting, few retrospective studies have reported short-lived efficacy of everolimus-based combination (with exemestane or fulvestrant) : median PFS was 4.9 months in N = 12 patients [20], 4.2 months in N = 41 patients [21] and 3.8 months in N = 79 patients [22]. These PFS were obtained in patients treated in 2^nd^ to 5^th^ lines and whose prior exposure to CDK4/6 inhibitors ranged from 4 to 21 months. Measurable disease according to RECIST was not a prerequisite for inclusion in these trials. Of note, in these retrospective reports, patients received everolimus as second or later line of therapy, which may explain the apparently shorter PFS compared to our prospective study. Finally, the fulvestrant and everolimus combination observed in our study appears somehow similar to the efficacy reported with fulvestrant and capivasertib combination: in the phase III CAPItello-291 trial (Patients were required to have RECIST measurable disease (version 1.1) or at least one lytic or mixed lytic–blastic bone lesion with identifiable soft-tissue components), the AKT inhibitor given in combination with fulvestrant led to a median PFS of 7.2 months and 5.5 months in patients with prior CDK4/6i exposure [23]. An appealing characteristic of everolimus is that its label allows using it in all patients, independently of their tumor mutational landscape. *PIK3CA* mutations have previously been reported to be associated with worse outcome in patients with ER + /HER2- mBC [24], while the efficacy of everolimus in clinical trial data is uncertain. In the neoadjuvant setting, Baselga et al. [25] found that the presence of *PIK3CA* exon 9 mutations conferred an improved response to the combination of everolimus with letrozole [25]. In various metastatic cancers, patients treated with PI3K/AKT/mTOR pathway inhibitors in combination with other treatments (ET, anti-HER2 therapy, or chemotherapy) had a longer time to progression compared to patients without *PIK3CA* mutations [26]. However, in the BOLERO-2 trial, the authors found no association between the presence of a *PIK3CA* mutation (either by next generation sequencing on tumor tissue or in cell-free DNA by ddPCR) and response to everolimus [27, 28]. In our study, we found that patients with *PIK3CA* mutation had a limited benefit, with a median PFS of 3.4 months and a median OS of 22.9 months. This median PFS appears to be shorter than reported with alpelisib in the BYLieve study [18] (7.3 months). The small sample size of this subpopulation in our study makes it difficult to draw definitive conclusions, but the impact of *PIK3CA* mutation on everolimus and fulvestrant efficacy after CDK4/6i needs to be further explored.

Finally, we investigated the impact of ctDNA dynamics as a predictive biomarker for the efficacy of everolimus plus fulvestrant. We found a prognostic impact of the baseline and 3–5 W ctDNA positivity on PFS and OS, and also a prognostic impact of the variation of ctDNA level between 3–5 W and baseline. However, among the patients with decreased ctDNA, three patients had a PD at the first radiological evaluation and two patients had a PR in spite of an increased ctDNA load. One hypothesis is that the mutations used for ctDNA monitoring in these patients were from a tumor subclone, which eventually couldn’t predict and represent the whole tumor evolution under treatment. Our results are in line with a recent study [29] monitoring ctDNA in fulvestrant-exemestane combination, where PFS was particularly short in patients with detectable ctDNA after 14 days of treatment: 2.1 months versus 5.0 months in those without detectable ctDNA (p = 0.012). A statistically significant longer PFS was observed for patients with ctDNA ratio (D14/baseline) below or equal to the median value (p ≤ 0.061): 4.4 months versus 1.9 months (HR = 2.5, 95%CI [1.0–6.2], p = 0.043). In the second line setting where multiple treatments are available and endocrine-resistance therapies are frequent, assessment of ctDNA dynamics during treatment could rapidly predict patients that will experience a tumor progression or response, but further studies are needed to assess its clinical utility. A phase 2 trial (“MONDRIAN”, NCT04720729) is underway to evaluate the use of early ctDNA level variation to monitor response to chemotherapy in HER2- mBC.

In conclusion, we report here the first prospective assessment of fulvestrant-everolimus in ER + /HER2- mBC patients who progressed on prior CDK4/6 inhibitor. We document that fulvestrant-everolimus is an active regimen in this population, with limited toxicity, whereas ctDNA detection demonstrated a satisfactory clinical validity for monitoring response to therapy.

## Methods

### Patients and blood sampling

All patients signed a written informed consent to this ethically approved study (*Comité de Protection des Personnes “Ile de France VI”*), registered with clinicaltrials.gov (NCT02866149, cohort #12). Eligibility criteria were: patients aged ≥18 years with ER + /HER2- mBC (immunoreactive for ER in ≥10% of tumor-cell nuclei by immunohistochemistry) treated at Institut Curie Hospitals (Paris and Saint Cloud), who progressed under prior CDK4/6i therapy (with no requirement for prior CDK4/6i duration). The presence of measurable disease (per RECIST criteria) was not a prerequisite for patient enrollment. All included patients were then treated with everolimus (10 mg/day, unless decided otherwise by physicians) and fulvestrant, per standard of care. Mucositis prophylaxis was left at the discretion of the physician. Tumor response to therapy was assessed at least every 3 months by CT-scan. The objective response rate (ORR) was calculated only with the patients with RECIST measurable disease, including those with unconfirmed responses. Any patient who did not achieve a complete/partial response or progressive disease (PD) within 6 months was considered as having a stable disease (SD). Adverse events were coded and graded according to the National Cancer Institute Common Terminology Criteria for Adverse Events (version 5.0). Patient characteristics and survival data were prospectively registered. Patients who discontinued everolimus treatment for any reason other than progression were required to follow the same schedule of assessments until progression. For each patient, longitudinal blood samples were collected: at baseline, after 3–5 weeks (3–5 W) of therapy and at time of disease progression. Variation in ctDNA was calculated using copies/ml.

### Plasma sample preparation, storage, DNA extraction and quantification

Blood samples were collected in EDTA tubes and plasma was isolated within 2 h by a two-step centrifugation: 820 × *g* for 10 min, then 16000 × *g* for 10 min at 4 °C. DNA was extracted using the QIAmp Circulating Nucleic Acid Kit (Qiagen, Hilden, Germany) according to the manufacturer’s instructions and quantified by Qubit 2.0 fluorometer (Invitrogen, Carlsbad, CA, USA) using the dsDNA HS Assay (Invitrogen, Carlsbad, CA, USA). Extracted DNA was stored at –20 °C before use.

### Identification of trackable somatic mutations

Archived tumor samples, either from the primary tumor or a metastatic deposit, were retrieved from the Pathology department. Tumor DNA was extracted from macro-dissected tumor tissue (Macherey-Nagel nucleospin tissue kit) and subjected to a large amplicon-based targeted NGS panel developed at Institut Curie, covering 483 genes (Qiagen Qiaseq). For patients with no tumor tissue available or failure of tissue DNA sequencing, targeted-NGS was performed on cfcDNA collected at baseline.

### Analysis of ctDNA by ddPCR

The identified somatic mutations were used for ctDNA monitoring in plasma by ddPCR. Tumor or synthetic DNA carrying the target mutation was used as positive control. Genomic DNA from healthy donors purchased from Promega (Madison, WI, USA) was used as negative control. Primers and probes for detecting *PIK3CA*: c.3140 A > G mutation are described in Supplementary Table 1. Assays for detecting *TP53*: c.637 C > T mutation was provided by SAGA Diagnostics (Lund, Sweden). ddPCR assays for detecting other mutations were purchased from Biorad laboratories (Hercules, CA, USA) (Supplementary Table 1). ddPCR assays were performed using the BioRad QX100 system (Biorad laboratories, Hercules, CA, USA) with 900 nM of each primer, 250 nM of each probe with one probe targeting the wide-type sequence and the other one targeting the mutant sequence being labeled with different fluorophores. The amplification was under the following conditions: 95 °C for 10 min (1 cycle); 40 cycles of 94 °C for 30 sec and hybridization at a specific temperature for each mutation for 60 s, and 98 °C for 1 min (1 cycle). The hybridization temperatures used for each assay were in Supplementary Table 1. Cluster thresholding and quantification were performed with QuantaSoft v.1.7.4 software. Droplets were manually assigned as wild-type (WT) or mutant (MUT) based on their fluorescence amplitude: WT: VIC+ or HEX + ; MUT: FAM + . The mutant allelic frequencies (MAFs) were calculated as follows: copy numbers of MUT / (copy numbers of WT + copy numbers of MUT). The total copy number of cfcDNA in each patient was calculated as the sum of the copy numbers of WT and MUT sequences. All experiments met the minimum requirements for digital PCR data [30, 31].

### ddPCR data analysis

The false-positive rate of each assay was estimated as previously reported [32, 33] using ≥ 15 replicates of WT DNA. The limit of blank (LOB) of different assays was defined as the upper 95% confidence limit of the mean false-positive measurements. Samples were considered as positive when MAF was higher than LOB with more than 1-3 positive droplets detected per analysis (Supplementary Table 1). When less than 300 amplifiable genomes were detected by ddPCR, the sample was not considered for further ctDNA analysis. The laboratory performing ddPCR experiments was blinded to patient outcomes.

### Statistical analysis

Categorical variables were compared using Fisher’s exact test. Survival analysis was performed using Kaplan–Meier plots with significance tested using the log-rank test. Progression-free survival (PFS), defined as the time from initiation of treatment to disease progression or death from any cause, was collected prospectively. Patients with no disease progression were censored at the last follow-up visit. OS was defined as the time from initiation of treatment to the date of death from any cause. Best overall response was defined as the best response recorded from the start of therapy until its discontinuation.

Cox proportional hazards modeling was used for univariate and multivariate analysis to evaluate the prognostic significance of different predictors. Baseline clinical and pathological characteristics with a p value > 0.1 in univariate analysis were excluded from multivariate analysis. Statistical analyses were performed with GraphPad Prism (version 8.0) or R software (version 4.1.1). P-values ≤ 0.05 were considered statistically significant.

### Supplementary information

Supplementary materials

Supplementary table 3

## Data Availability

The human sequence data underlying this article cannot be shared due to patient privacy. Other data generated in this study are available within the article and its supplementary data files.
