# Supplementary material for: Fulvestrant and everolimus efficacy after CDK4/6 inhibitor: a prospective study with circulating tumor DNA analysis
Source: Oncogene. 2024 Feb 27;43(16):1214–22. doi: 10.1038/s41388-024-02986-6 (PMC11014798; doi:10.1038/s41388-024-02986-6)
Supplement: Supplementary file 1 — Supplementary materials [file 41388_2024_2986_MOESM1_ESM.docx]

**Supplementary materials**

**
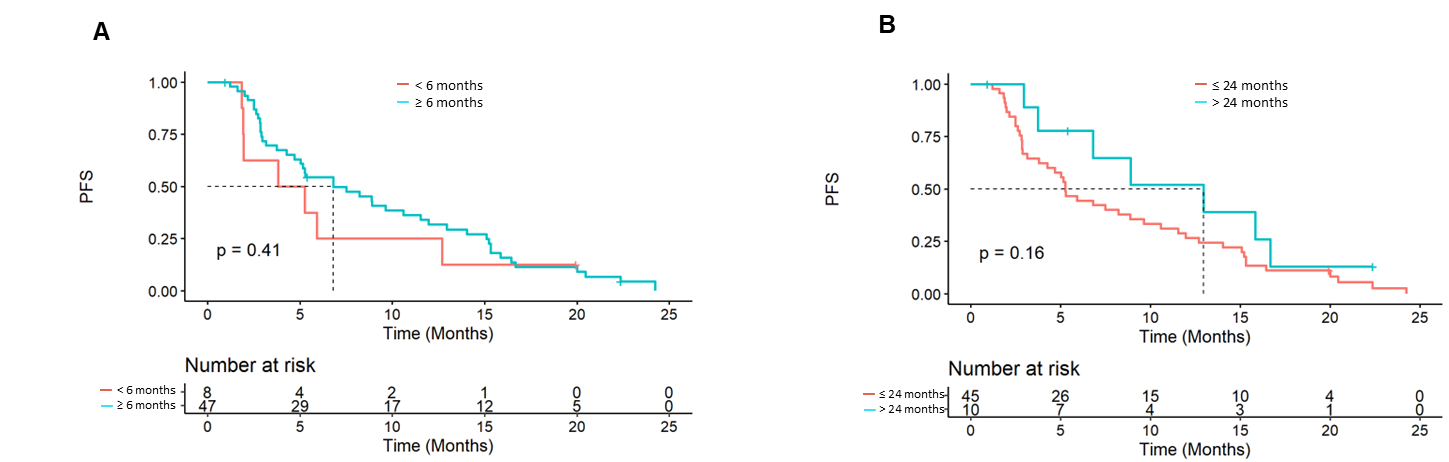
**

**Supplementary figure 1**. PFS according to median PFS of palbociclib: < 6 months (A) or > 24 months (B).

PFS: progression-free survival.


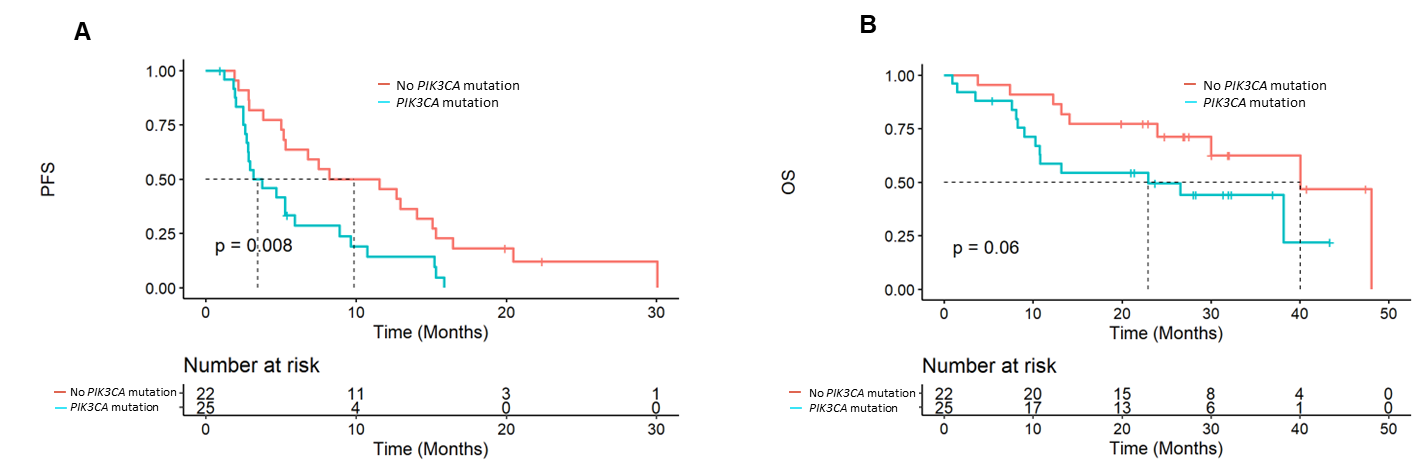


**Supplementary figure 2**. Progression-free survival (A) and overall survival (B) according to *PIK3CA* mutation.

PFS: progression-free survival; OS: overall survival.

**
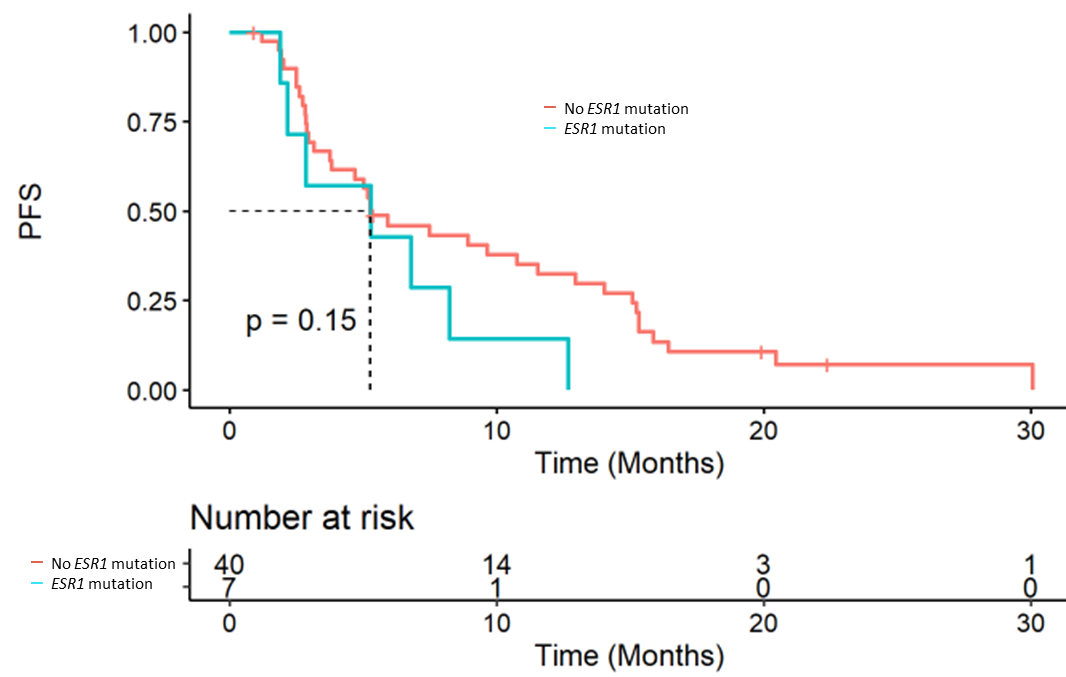
**

**Supplementary figure 3**. Progression-free survival according to *ESR1* mutation.

PFS: progression-free survival.

**
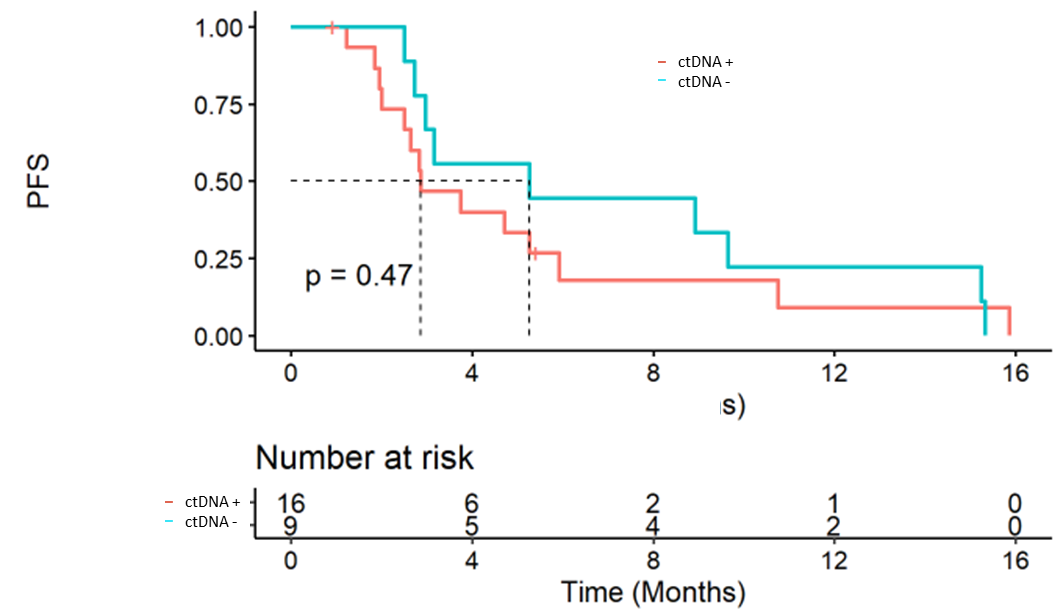
**

**Supplementary figure 4**. Progression-free survival among patients with *PIK3CA* mutation according to ctDNA positive or negative.

PFS: progression-free survival.

**Supplementary table 1**. ddPCR assays.

| **Mutation targeted by ddPCR** | **Provider** | **Reference number** | **LOB** | **Ta** | **Minumum droplet** |
| --- | --- | --- | --- | --- | --- |
| AKT1 : c.49G>A | Biorad | dHsaMDV2010031 | 0.036 | 55 | >3 |
| APC : c.1786T>C | Biorad | dHsaMDS702205622 | 0.000 | 55 | >1 |
| ATM : c.866_868del | Biorad | dHsaMDS531312748 | 0.000 | 55 | >1 |
| CUX1: c.3556C>T | Biorad | dHsaMDS846834797 | 0.000 | 55 | >1 |
| ESR1 : c.1610A>C | Biorad | dHsaMDS975379796 | 0.000 | 55 | >1 |
| ESR1 : c.1613A>G | Biorad | dHsaMDS460485301 | 0.006 | 55 | >2 |
| ESR1: c.1605_161del | Biorad | dMDS140108909 | 0.000 | 55 | >1 |
| GATA3 : c.1220dup | Biorad | dHsaMDS804009364 | 0.000 | 55 | >1 |
| GATA3 : c.998delinsGT | Biorad | dHsaMDS404204051 | 0.006 | 55 | >2 |
| KRAS: c.35G>T | Biorad | dHsaMDV2510592 | 0.000 | 55 | >1 |
| PI3KCA: c.2597T>G | Biorad | dHsaMDS319038639 | 0.000 | 55 | >1 |
| PIK3CA : c.1035T>A | Biorad | dHsaMDS2516778 | 0.000 | 55 | >1 |
| PIK3CA : c.1633_1634delinsAG | Biorad | dHsaMDS372838164 | 0.000 | 55 | >1 |
| PIK3CA : c.1633G>A | Biorad | dHsaMDV2010075 | 0.033 | 55 | >3 |
| PIK3CA : c.1634A>G | Biorad | dHsaMDS175985942 | 0.022 | 55 | >2 |
| PIK3CA : c.3140A>G | Thermofisher |  | 0.014 | 60 | >2 |
| PIK3CA : c.3193_3202delinsTT | Biorad | dHsaMDS487937044 | 0.000 | 55 | >1 |
| PIK3CA: c.1268C>T | Biorad | dHsaMDS122023940 | 0.017 | 55 | >2 |
| PIK3CA: c.197C>T | Biorad | dHsaMDS503667651 | 0.017 | 55 | >2 |
| PIK3CA: c.1357G>C | Biorad | dHsaMDS256883370 | 0.000 | 55 | >1 |
| PIK3CA: c.3140A>T | Biorad | dHsaMDV2010123 | 0.000 | 55 | >1 |
| PTEN: c.955_958del | Biorad | dHsaMDS417315727 | 0.000 | 55 | >1 |
| RET: c.2731 G>A | Biorad | dHsaMDS887659414 | 0.000 | 55 | >1 |
| TP53 : c.216del | Biorad | dMDS858067005 | 0.014 | 55 | >1 |
| TP53 : c.488A>G | Biorad | dHsaMDV2010099 | 0.000 | 55 | >1 |
| TP53 : c.517G>A | Biorad | dHsaMDV2510514 | 0.000 | 55 | >1 |
| TP53 : c.637C>T | SAGA | ATR1100P53A004 | 0.018 | 72 and 48 | >2 |
| TRRAP : c.6883C>T | Biorad | dMDS436062977 | 0.010 | 55 | >2 |

| **PIK3CA : c.3140A>G** | **Sequence** |
| --- | --- |
| Primer F | GGCTTTGGAGTATTTCATGAAACA |
| Primer R | GAAGATCCAATCCATTTTTGTTGTC |
| Probe1 (FAM - MGB) | TGATGCACGTCATGGT (mut) |
| Probe 2 (VIC - MGB) | ATGATGCACATCATGGT (WT) |

**Supplementary table 2**. Tumor response with everolimus plus fulvestrant.

| **Response** | **Number**  **of patients**  **N=47** | **%** |
| --- | --- | --- |
| **Best overall response** |  |  |
| Complete response | 0 | 0 |
| Partial response | 15 | 31.9 |
| Stable disease $\geq$ 6 months | 11 | 23.4 |
| Progressive disease | 21 | 44.7 |
| **6 months clinical benefit rate** | 26 | 55.3 |

**Supplementary table 4**. Univariate analysis for progression-free survival and overall survival.

| **Cox univariate analysis** | | | | |
| --- | --- | --- | --- | --- |
|  | **Progression-free survival** | | **Overall survival** | |
| **Variable** | **HR (95%CI)** | ***p* value** | **HR (95%CI)** | ***p* value** |
| Age | 1.00 (0.98-1.00) | 0.83 | 1.00 (1.00-1.10) | **0.03** |
| Tumor grade | 0.71 (0.33-1.50) | 0.38 | 0.71 (0.24-2.10) | 0.54 |
| Synchronous metastases | 1.30 (0.72-2.20) | 0.42 | 1.30 (0.61-3.00) | 0.47 |
| Neo/adjuvant chemotherapy | 0.74 (0.42-1.30) | 0.30 | 1.20 (0.54-2.60) | 0.68 |
| Number of metastatic sites | 1.60 (0.90-2.80) | 0.11 | 1.30 (0.57-2.80) | 0.56 |
| Only bone metastases | 0.55 (0.28-1.10) | 0.08 | 0.96 (0.38-2.50) | 0.93 |
| Visceral metastases | 1.30 (0.71-2.20) | 0.44 | 0.77 (0.35-1.70) | 0.53 |
| Performance status | 1.00 (0.53-1.90) | 0.98 | 2.90 (1.20-6.90) | **0.02** |
| Prior number of lines | 0.89 (0.43-1.80) | 0.74 | 1.40 (0.50-3.70) | 0.54 |
| Palbociclib treatment duration | 0.83 (0.47-1.40) | 0.50 | 0.84 (0.37-1.90) | 0.68 |
| *PIK3CA* mutation | 2.30 (1.20-4.40) | **0.01** | 2.20 (0.92-5.40) | 0.07 |
| Baseline ctDNA detection | 2.04 (1.20-3.45) | **0.008** | 3.85 (1.39-10.99) | **0.01** |
| 3-5 weeks ctDNA detection | 2.38 (1.33-4.17) | **0.004** | 3.45 (1.52-7.69) | **0.003** |
| Clearance of ctDNA at 3-5 weeks | 0.36 (0.13-0.99) | **0.05** | 0.31 (0.09-1.10) | 0.08 |
| ctDNA decrease at 3-5 weeks | 0.15 (0.06-0.41) | **0.0002** | 0.27 (0.09-0.79) | **0.02** |
| F ctDNA level (high or low) * | 1.40 (0.69-3.00) | 0.33 | 0.62 (0.25-1.50) | 0.30 |

HR: hazard ratio; CI: confidence interval; vs: versus. * Only in patients with ctDNA detection and median copy number used as a threshold.
